# Investigating the Performance of the Multi-Lobed Leaf-Shaped Oscillatory Obstacles in Micromixers Using Bulk Acoustic Waves (BAW): Mixing and Chemical Reaction

**DOI:** 10.3390/mi14040795

**Published:** 2023-03-31

**Authors:** Vahid Kordzadeh-Kermani, Hossein Dartoomi, Mina Azizi, Seyed Nezameddin Ashrafizadeh, Masoud Madadelahi

**Affiliations:** 1 Research Lab for Advanced Separation Processes, Department of Chemical Engineering, Iran University of Science and Technology, Tehran 16846-13114, Iran; v_kordzadeh@chemeng.iust.ac.ir (V.K.-K.);; 2 Department of Electronics, South Tehran Branch Azad University, Tehran 15847-15414, Iran; 3 Department of Mechanical Engineering, Isfahan University of Technology, Isfahan 84156-83111, Iran; 4 School of Engineering and Sciences, Tecnologico de Monterrey, Monterrey 64849, NL, Mexico

**Keywords:** microfluidics, active micromixers, acoustic streaming, bulk acoustic wave, chemical reaction, mixing efficiency

## Abstract

Proper mixing in microfluidic devices has been a concern since the early development stages. Acoustic micromixers (active micromixers) attract significant attention due to their high efficiency and ease of implementation. Finding the optimal geometries, structures, and characteristics of acoustic micromixers is still a challenging issue. In this study, we considered leaf-shaped obstacle(s) having a multi-lobed structure as the oscillatory part(s) of acoustic micromixers in a Y-junction microchannel. Four different types of leaf-shaped oscillatory obstacles, including 1, 2, 3, and 4-lobed structures, were defined, and their mixing performance for two fluid streams was evaluated numerically. The geometrical parameters of the leaf-shaped obstacle(s), including the number of lobes, lobes’ length, lobes’ inside angle, and lobes’ pitch angle, were analyzed, and their optimum operational values were discovered. Additionally, the effects of the placement of oscillatory obstacles in three configurations, i.e., at the junction center, on the side walls, and both, on the mixing performance were evaluated. It was found that by increasing the number and length of lobes, the mixing efficiency improved. Furthermore, the effect of the operational parameters, such as inlet velocity, frequency, and intensity of acoustic waves, was examined on mixing efficiency. Meanwhile, the occurrence of a bimolecular reaction in the microchannel was analyzed at different reaction rates. It was proven that the reaction rate has a prominent effect at higher inlet velocities.

## 1. Introduction

The process of mixing is known as a crucial subject in microfluidic devices for different purposes. Many processes in microdevices require homogenous, facile, and quick mixing which guarantees proper performance for applications such as nanoparticle synthesis, drug delivery, polymerization, assaying, diagnosis, reaction engineering, etc. [1,2,3,4,5]. Due to the low fluid flow velocity and micrometric dimensions of microfluidic devices, very low Reynolds numbers and the laminar fluid regime are dominant in such devices. Consequently, the diffusion mechanism is responsible for the mass transfer and transporting of solute substances in the microfluidic systems, which prolongs the mixing time for a highly efficient mixing process. [6,7]. Necessarily, utilizing active or passive mixers is an inevitable subject in respective microfluidic devices to enhance their efficiency. Passive micromixers generally create perturbations and hydrodynamic interactions in the fluid flow by placing modifications such as obstacles on microchannel geometry. The zigzag-shaped, serpentine-shaped, T-shaped, z-connection, tree-like, and bend/expansion geometries are popular examples of passive micromixers extensively investigated by researchers [8]. Despite having advantages enumerated for passive micromixers, such as simplicity, low-cost fabrication and convenient maintenance, they suffer from some drawbacks, including significant pressure drop, clogging, need for extra pumping, and limited production rates [9]. To this end, applying active (moving) external parts can enhance the mixing efficiency in microfluidic devices. The active micromixers integrated into microfluidic devices include mainly magnetic, electrokinetic, optical, thermal, and acoustic modules fortifying the mixing process by imposing external perturbations from outside energy sources [10,11].

Modulating an acoustic source with a microfluidic device will provide the media for propagating the acoustic waves in the fluid, leading to the emerging acoustofluidic concept [12]. Acoustic waves can be produced in two categories: surface acoustic waves (SAW) and bulk acoustic waves (BAW), which differ in generation source. The SAWs are generally produced from interdigitated electrodes (IDT) on a piezoelectric substrate and propagated along the interface surface of the microchannel and a piezoelectric thin film (Lithium niobate LiNbO_3_ substrate) [13,14]. On the contrary, the BAWs produced from detachable piezoelectric transducer parts propagate to the whole body of the fluid [15]. The idea of integrating the acoustic waves for moving particles/fluids is taken from the work of Rayleigh and Hutchisson [16,17]. Accordingly, the concept of the acoustic streaming (AS) phenomenon has emerged, representing a steady flow generated based on hydrodynamics and acoustics interaction. Researchers utilized acoustic waves in a microchannel for the early applied attempts to enhance the mixing process in different geometries [18,19]. The use of AS in microfluidic devices was continued in various studies for a vast range of applications such as particles manipulation/migration, mixing of fluids, synthesize of micro/nanoparticles, microfluidic pumps, etc., where the mixing process using an active mixer has become of great importance [20,21,22,23,24]. Active micromixers have the ability to provide a perfect mixing and uniform distribution of substances in a short time at the minimum distance of a microfluidic device.

The AS phenomenon considerably enhances the transport of substances through the fluid flow by fortifying the mixing intensity via breaking the laminar flow interfaces and producing vortices. Integrating the AS with microfluidic devices for mixing approaches can be performed using two methods: oscillating the micro solid structures (i.e., sharp edges) or oscillating microbubbles.

Recent studies in the field of acoustic streaming (mixing) into microfluidic devices can be classified into the categories of experimental-based, numerical-based, and also a combination of them [25]. One of the first attempts can be attributed to the work of Frampton et al., in which acoustic streaming in various-sized microchannels was analyzed mathematically for different types of stagnant fluids [26]. Some researchers preferred to utilize sharp-edge geometries as oscillatory parts in microdevices. In this regard, implementing the saw-tooth-shaped structure on the walls of a microchannel to study the mixing of two fluid flow was extensively analyzed [27,28]. The formation of vortices around the tips of triangular shapes due to acoustic oscillations was observed in the experiments, which were approved numerically and analyzed for various structures. [29].

Some researchers studied more complex sharp-edged designs and modified them to evaluate the effect of this alteration. For instance, the Tesla structure [30], multi-edge structures (such as flower-shaped) [31], and lotus-shaped designs [32] were utilized in highly efficient controllable mixing devices. Conversely, some researchers preferred simple designs for their sharp-edge acoustic micromixers [33]. In this regard, Endaylalu and Tien designed and studied a T-junction comprised of a triangular sharp edge located at the junction numerically and experimentally [34,35].

**Figure 1:**
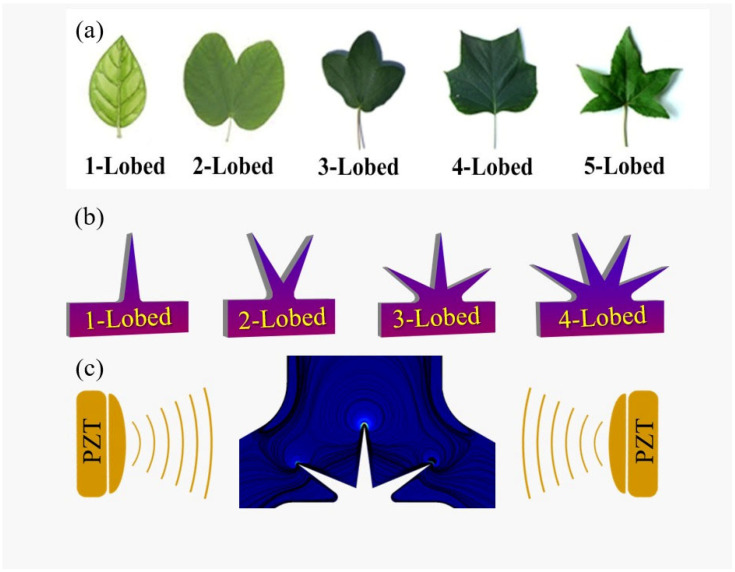
(**a**) The illustration of various types of multi-lobed leaves structures exploited for designing the leaf-shaped oscillatory sharp-edges. (**b**) The schematic structure of oscillatory obstacles exploited in this study. (**c**) The illustration of piezoelectric actuators (PZT). The vibration of the piezoelectric causes the oscillatory movement of sharp-edge lobes and generates multiple vortices.

Based on the mentioned discussion, it can be inferred that the simple design of sharp edges and more efficient structures are preferable items for providing efficient mixing in microfluidic devices. Therefore, the main objective of this study is first defined as designing and numerically studying an acoustic micromixer comprising a Y-junction having multi-lobed leaf-shaped sharp edges (as nature-inspired structures, see Figure 1) located at different places in the microchannel. In this regard, different structures of multi-lobed obstacles, including 1, 2, 3, and 4-lobed geometries, are assigned as oscillatory obstacles. The effect of these structures is simulated and analyzed on mixing performance. Additionally, the geometrical characteristics of obstacles, including lobes’ lengths, lobes’ inside angle, and lobes’ pitch angle, are evaluated along with the goal of finding the optimum placement of the obstacles in the microchannel. Furthermore, the effect of operational parameters such as inlet flow rate, frequency of acoustic waves, and displacement amplitude on mixing performance is numerically investigated. Secondly, we evaluated the performance of the acoustic microchannel by considering implementing a bimolecular elementary chemical reaction at different rate constants and operational conditions.

## 2. Numerical Procedure

### 2.1. Defining the System’s Geometry and Scheme

**Figure 2:**
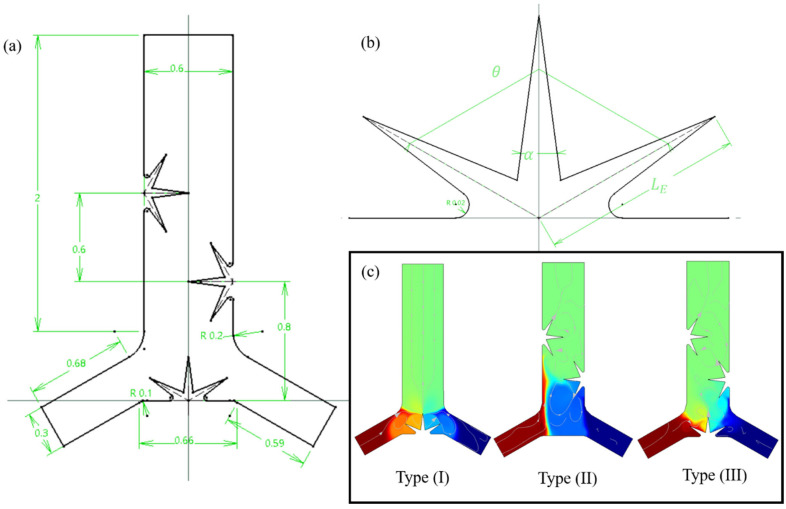
(**a**) A schematic illustration of an acoustic micromixer along with dimensions annotation. (**b**) The illustration of a 3-lobed leaf-shaped obstacle and the definition of parameters: length ($L_{E}$), pitch angle (θ), and inside angle (α). (**c**) Three types of configurations for oscillatory obstacle placement inside the microchannel; Type (I) at the center of the junction; Type (II) on the side walls oppositely and asymmetrically; type (III) a combination of types (I) and (II).

An acoustic microchannel with a length of 2 mm and a width of 0.6 mm is defined in this study as a Y-shaped junction with two inlets and an outlet into which the leaf-shaped oscillatory obstacle(s) are placed. Two distinct fluids with different concentrations of solute substances enter from inlets using a pressure-driven method in a laminar flow mode, which maintains an isothermal condition. We considered two situations of mixing, i.e., without chemical reaction and with chemical reaction. The acoustic waves (BAWs) generated from the piezoelectric transducer placed beside or beneath the microchannel impose oscillatory vibrations on sharp-edges (lobes). The particular geometry of the oscillatory leaf-shaped sharp-edges produces vortices around the lobes’ tips in the body of the fluid flow. A schematic representation of the system geometry with the relative dimensions is represented in Figure 2a. A typical 3-lobed sharp edge is depicted in Figure 2b annotating the parameters of lobes length ($L_{E}$), lobes inside angel (α), and lobes pitch angle (θ). Additionally, we exploited three different configurations for placing the leaf-shaped obstacles inside the microchannel. As illustrated in Figure 1c, the Type I configuration contains an oscillatory obstacle that is placed at the center of the junction; Type II contains two opposite asymmetrical obstacles, and Type III represents a combination of Types I and II (Figure 2c).

### 2.2. Theory 

Applying acoustic waves with their harmonic nature in a microfluidic system can also impose much complexity on the numerical solutions. Accordingly, Nyborg proposed a perturbation method for such problems in which the harmonic actuations were divided into two parts: a harmonic component and a time-averaged second-order component [36]. As such, there are three types of velocity that can be defined by employing this theory, i.e., zeroth-order, first-order, and second-order velocities. The details of perturbation theory equations are extensively elaborated in the references [27,37]. The details of equations solved in this numerical study are attached in the Supplementary Document.

Additionally, the mixing index (MI), which represents the efficiency of mixing at each cross section along the channel length, is calculated using Equation (1) ($C_{i}$: concentration of solute substances along the cross-section line at a fixed distance from the microchannel inlet, $C_{avg}$: average concentration along the cross-section line, and n: number of the sampled points).

(1) $$MI=\frac{\sqrt{\frac{\sum {\left(C_{i}-C_{avg}\right)}^{2}}{n-1}}}{C_{avg}}.$$

## 3. Simulation Methodology

### 3.1. System’s Boundary Conditions

In Table 1, the main boundary conditions exploited for solving the problem are provided. 

### 3.2. Numerical Implementation

The differential equations representing the system were solved using the finite element method. The parameters used in the simulation are listed in Table 2. We used the following steps to implement the simulation:

i. The first-order equations were solved using the “Thermoviscous acoustic” module by considering boundary conditions.
ii. The zeroth-order and second-order equations were solved using the “Laminar flow” module by applying weak contribution modification for some equations.
iii. The equations for concentration were solved using the module for “Transport of Diluted Species”.

**Table 2:** The physical characteristics and operational parameters used in the simulation study [25,31,35,38,39].

	Parameter	Description	Value(s)
Physical characteristics of the system	$\rho_{0}$	Density of fluid	997 kg/m^3^
	$c_{0}$	Speed of sound in water	1497 m/s
	$\mu_{B}$	Viscosity of fluid (Bulk)	2.47 mPa s
	μ	Shear viscosity	0.890 mPa s
	$k_{0}$	Compressibility	4.48 × 10^−10^ Pa^−1^
	$C_{p}$	Specific heat capacity	4180 J/kg.K
	$D_{th}$	Thermal diffusivity	1.464 × 10^−7^ m^2^/s
	$k_{th}$	Thermal conductivity	0.61 w/(m.K)
	$\alpha_{th}$	Thermal expansion coefficient	2.74 × 10^−4^ 1/K
	γ	Specific heat capacity ratio	1.012
	T	Absolute temperature	298.15 K
	$D_{i}$	Mass diffusion coefficient of solute	4 × 10^−10^ m^2^/s
Operational parameters	$d_{0}$	Oscillation amplitude (displacement)	1.4 to 3 µm
	$Log\;\left(k\right)$	Kinetic constant of the chemical reaction	−2 to 1
	$L_{E}$	Lobe’s length	200 to 350 µm
	$v_{in}$	Inlet velocities (background velocity)	50 to 400 µm/s
	α	lobes’ side inside angel	15° to 45°
	θ	lobes’ pitch angle	60° to 120°
	$f_{0}$	Actuation frequency	4 to 10 kHz

### 3.3. Model Verification and Setting up the Numerical Procedure

To validate our numerical procedure and its results, we used the available data in the literature from the works of Nama et al. and Endaylalu and Tien [27,35]. In this regard, identical geometries were designed, and by adjusting an identical operational parameter, the results of the simulation were compared to related data. To this end, the mixing index at various cross-sections was calculated. Through this study, a reasonable deviation from experimental data was calculated (Figure S1a,b). The results of our validation study showed good consistency with the literature results. Additionally, a mesh-independent study was performed by considering various numbers of cells and evaluating the mixing index. As shown in Figure S2, a minimum of about 15,000 cells is a reasonable choice in Type III geometry for different multi-lobed structures, i.e., at higher numbers, there is no remarkable change that will appear in the mixing index value. Therefore, the remaining simulations were performed by considering this number of cells for geometries.

In this study, we first optimized the shape of leaf-shaped obstacles for the Type I and Type II configurations. Following that, the mixing index for various inlet flow rates, oscillation frequencies, and displacement amplitude was analyzed.

## 4. Results and Discussions

**Figure 3:**
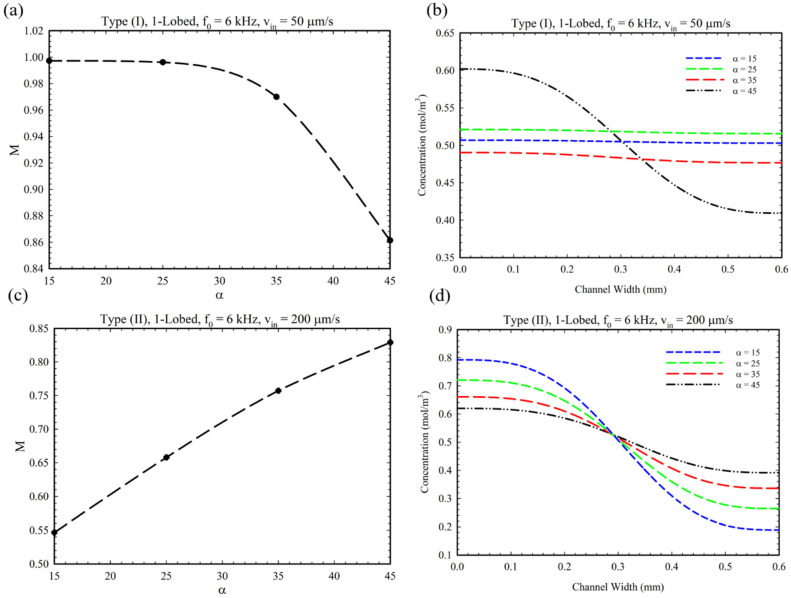
MI and concentration profiles for different α values: (**a**,**b**) Type I and (**c**,**d**) Type II configuration.

Figure 3 illustrates the effect of the lobes’ side interior angle for Type I and Type II geometries. As is demonstrated in Figure 3a, by increasing the value of the α from 15° to 45°, the mixing index decreased by 15%. This is because by increasing the value of α, the lobe’s base and width grow, and the tip’s movement will be restricted. Therefore, by limiting the oscillatory movement of the tip, the number and the power of the formed vortices decreased, which lowered the mixing index parameter in Type I geometry. In addition, a wider triangular base at the junction limits the area through which the inlet fluids can pass, thereby reducing the MI parameter. By indicating the concentration profiles of solute substances at the outlet cross-section of the microchannel, Figure 3b demonstrates the decrease in the mixing index. In contrast to Type I, in Type II geometry, increasing the parameter α increases the mixing index by approximately 50% (Figure 3c,d). This observation can be explained by the fact that the two opposing, asymmetric obstacles impose a synergistic effect on their placement and the vortices they generate, which increases the mixing index by increasing the α parameter.

**Figure 4:**
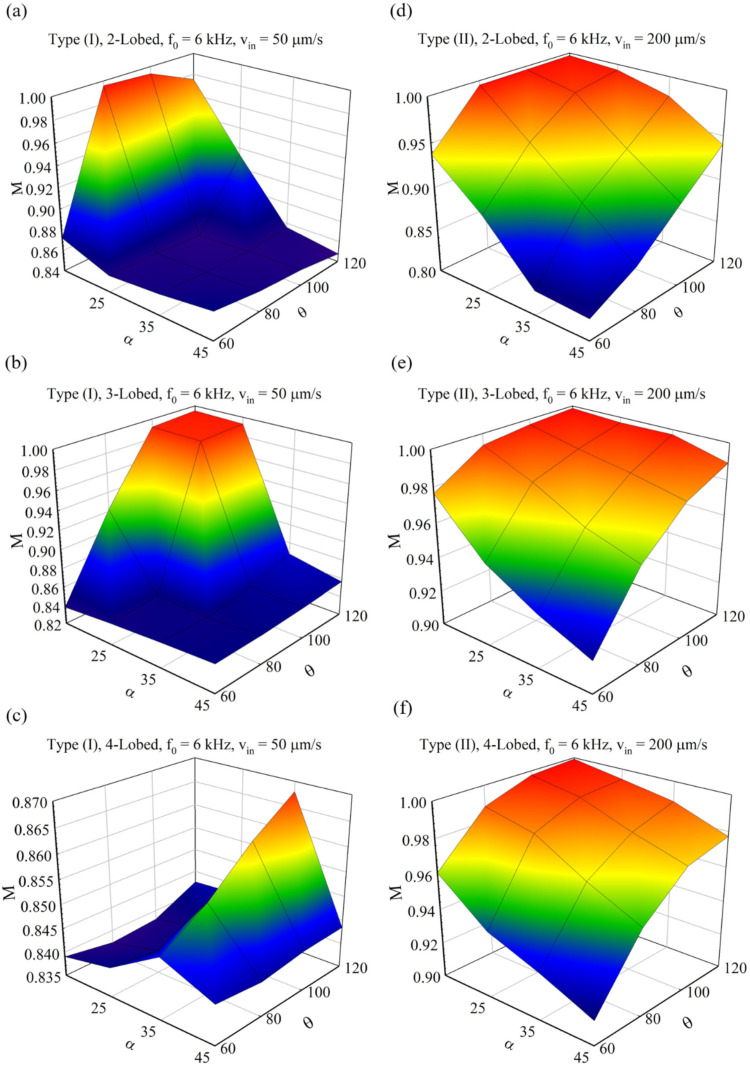
MI parameter with respect to angles α and θ of obstacles: (**a**–**c**) Type (I) for 2, 3, and 4-lobed structure. (**d**–**f**) Type (II) for 2, 3, and 4-lobed structure.

In these systems, each sharp edge produces a vortex around itself since each lobe is vibrated independently by the acoustic agent, and increasing the number of lobes will enhance mixing. Yet, when two lobes are located near each other, a type of interaction is produced in the vortices; hence, the angle between the lobes θ (pitch angle) can play a significant role in enhancing the MI of these systems. Figure 4 shows the 3D diagrams of the changes in the MI for types (I) and (II) based on the changes in the angles of α and θ. Since θ is not applicable for 1-lobed structures, these diagrams are prepared for 2, 3, and 4-lobed items.

Accordingly, based on the results obtained in Figure 3 and Figure 4, we acquired the best value for angles α and θ for further investigations. For this, the optimal angles used in the continuation of the modeling are listed in Table 3.

As can be seen in these figures, generally in Type (II), a higher average MI is achievable using different combinations of these two angles. In the case of 2-lobed structures, the generated vortices overlapped and may increase/decrease the mixing index parameter; therefore, the θ parameter is of great importance. 

**Figure 5:**
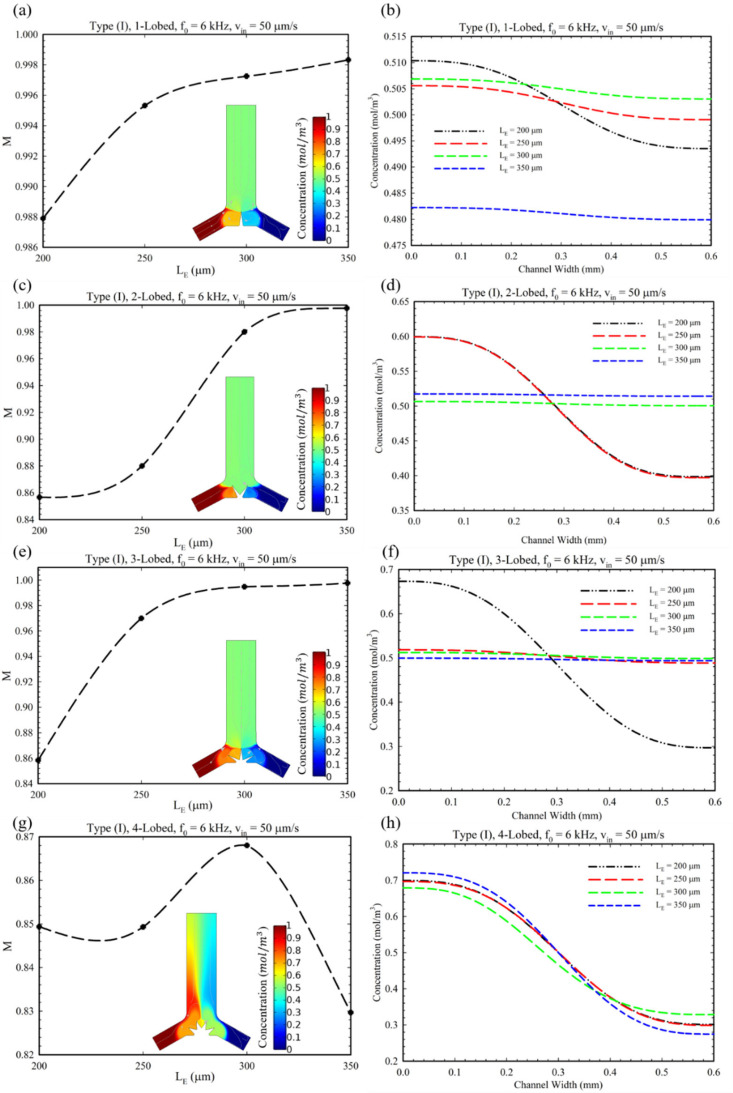
(**a**,**c**,**e**,**g**) The diagram of changes in MI for different lengths ($L_{E}$) for Type I, II, and III configurations and multi-lobed structures. (**b**,**d**,**f**,**h**) The concentration profile of solute at the outlet of microchannel for different multi-lobed structures.

Figure 5 shows the effect of changes in the number of lobes and their length ($L_{E}$) on the MI. In this regard, as each lobe in the oscillatory situation can generate counter-rotating vortices, by increasing the number of lobes, the number of generated vortices and, accordingly, the MI will be increased [31]. However, in the case of the 4-lobed structure, the mixing index has a lower quantity in comparison with other structures in this configuration (Type I). This observation can be explained by the reason that by increasing the lobes up to four, weaker vortices are generated, and they annihilate the effect of each other. Additionally, in the case of =350 μm, the MI decreased since this length of lobes hinders the fluid entrance to the microchannel; therefore, the MI is eventually reduced. Accordingly, 300 μm was defined as an optimum length for this configuration (Type I). Furthermore, as shown in Figure 5b for the length of $L_{E}$ = 350 µm, it is observed that the mean of the concentration profile has a value lower than other lengths (≈0.48). This observation is due to the fact that by having a longer length and sharper tip, stronger vortices are generated. Consequently, the stronger generated vortices generate backflows which prevent the passages of proper fluid flow and solute (see Figure S3a). Therefore, a decrease in concentration profile for the length of $L_{E}$ = 350 µm at the outlet is observed. 

**Figure 6:**
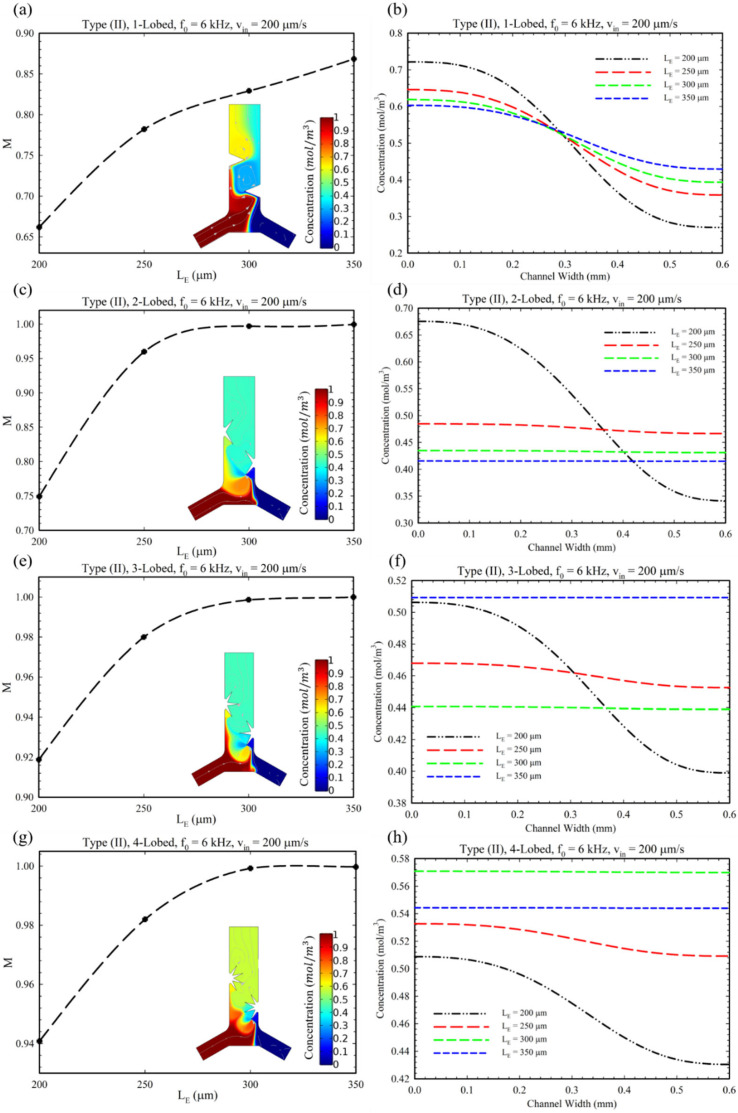
The diagram for analyzing the performance of microchannel Type II configuration having different multi-lobed structures: (**a**,**c**,**e**,**g**) the effect of length ($L_{E}$) of lobes on MI. (**b**,**d**,**f**,**h**) The concentration profile of solute substance at the outlet cross-section for Type II.

Figure 6 shows the changes in the MI for different values of lobe length ($L_{E}$) and the concentration profile at the microchannel outlet cross-section corresponding to the Type (II) configuration. As the MI diagram shows, increasing the length ($L_{E}$) improves the mixing for Type (II) microchannels for all multi-lobe structures. Considering the fact that the excessive increase in the length of the lobes prevents the passage of fluid (Figure 5, 4-lobed structure), we recommend using lobes with a length of 300 μm in this type of microchannel. Furthermore, in the case of the 1-lobed structure, it is presented that the mixing index has not touched values higher than 0.85. This fact is related to two reasons: (1) The more lobes acoustically vibrated under acoustic actuation, the more vortices are generated around the tips. Consequently, the 1-lobed structure has the lowest possible lobes, which acquires the lowest number of vortices generated, and the mixing index remains fixed in the range of 0.65 to 0.85. (2) The sharpness of sharp-edges plays a key role in generating the vortices, i.e., the sharper the edge, the more powerful vortices are generated, which can mix two fluids more efficiently [24]. In Figure 6d,f, it is observed for $L_{E}$’s value higher than 200 µm, a negative deviation from the mean of concentration is recognized. There are two reasons that can be considered for this issue. Firstly, by increasing the length of the lobes in this situation, the passage of the fluid is limited, and the fluid flow reduces. Secondly, by increasing the $L_{E}$, stronger vortices are produced, which hinder the inlets. Accordingly, for these reasons, a limited flow rate from the left inlet is introduced in comparison to $L_{E}$ = 200 µm, which reduces the concentration profile of the solute (see Figure S3e,h). Adversely, for Type II, 4-lobed structure (Figure 6h), a positive deviation from the mean value of concentration is observed. This can also be attributed to the weaker vortices, which allow entering more solute from the left inlet to the microchannel and act as a pumping action for the left fluid flow (see Figure S3k).

**Figure 7:**
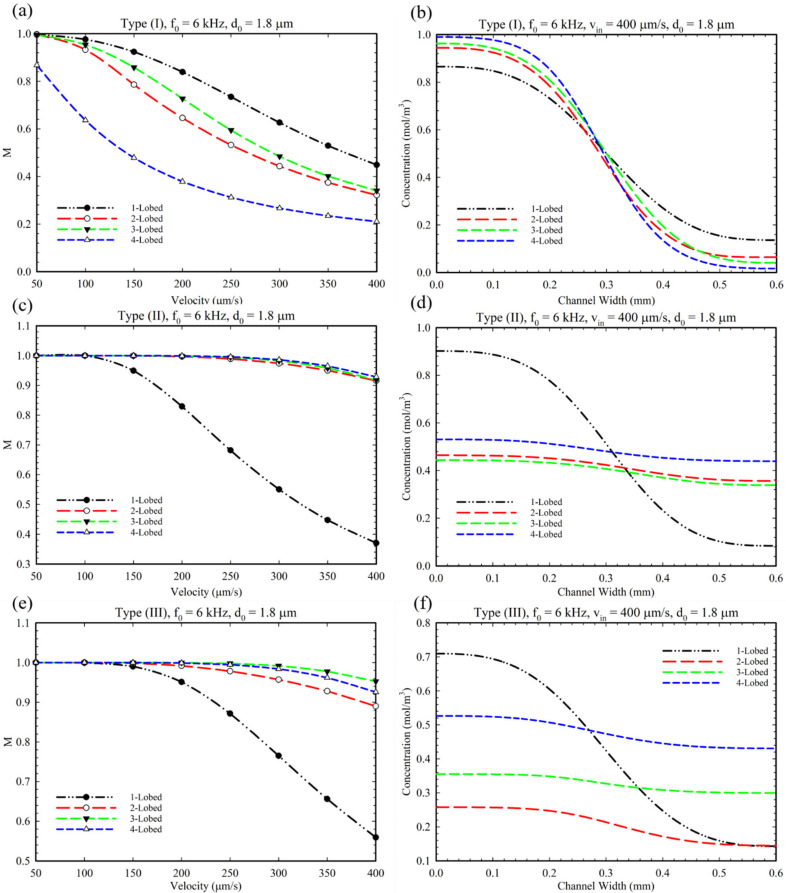
The diagram of the effect of inlet velocity on MI. (**a**,**c**,**e**) for type I, II, and III for multi-lobed structures. (**b**,**d**,**f**) The corresponding concentration profile at the outlet cross-section.

As mentioned in the introduction, the Type III configuration is defined with a combination of Type I and II, i.e., having oscillatory obstacles both at the junction’s center and side walls. In order to analyze this type of obstacle’s configuration, we exploited the optimized parameters (α, θ, $L_{E}$) from later sections for this section. Accordingly, the effect of changing the inlet velocity on the MI is evaluated based on previous optimized results. Figure 7 presents the effect of increasing the inlet velocity on MI for Type I, II, and III configurations. From this figure, it can be inferred that, by increasing the inlet velocity, the MI decreased due to the dominancy of fluid flow over the acoustic streaming and mixing. Additionally, as is illustrated in Figure 7f, for the 2 and 3-lobed modes, the average solute concentration decreased to the value of 2 and 3.1, respectively. This decrease in concentration can be attributed to the vortices around the tips and their confluence of them (see Figure S3f,i), which obstructed the left inlet (inlet for solute) from having a proper flow rate. Therefore, a lower amount of fluid and solute passes through the left inlet, and consequently, a decrease in concentration is observed at the outlet.

**Figure 8:**
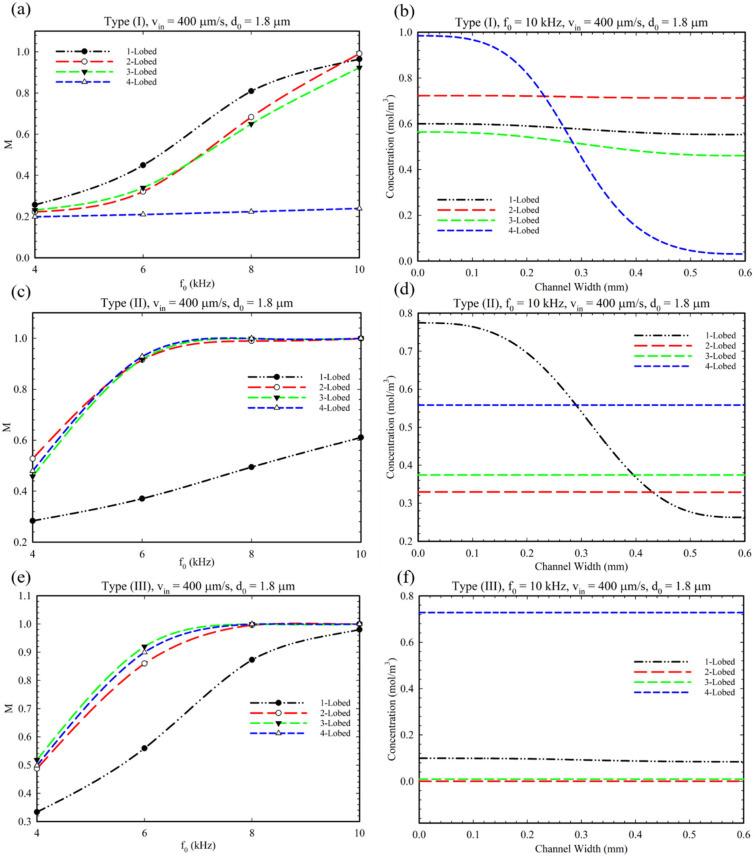
The diagram of changes in the MI based on frequency change in the microchannel. (**a**,**c**,**e**) The effect of frequency intensity on mixing index for Type (I), Type II, Type III, and multi-lobed structures. (**b**,**d**,**f**) The concentration profile at the outlet of microchannel at 10 kHz frequency.

In Figure 8, we analyzed the effect of frequency changes on the MI and local concentration profile at the outlet for all geometries and microchannel Types (I), (II), and (III). As can be seen in the graphs, increasing the applied frequency increases the MI in all three types of microchannels and all geometries. Figure 8b shows that the increasing frequency has no remarkable effect on the mixing index of the 4-lobed structure (Type I) because of small and weak vortices produced in this mode (see Figure S3j). As mentioned in the literature, higher frequencies can enhance MI [29]. As shown in Figure 8f, at high frequencies (≈10 kHz), almost no solute can pass through the left inlet due to the high intensity of the generated vortices. Therefore, the concentration profiles have a low and fixed value for 3 and 4-lobed structures despite having a proper MI value (Figure 8e). 

**Figure 9:**
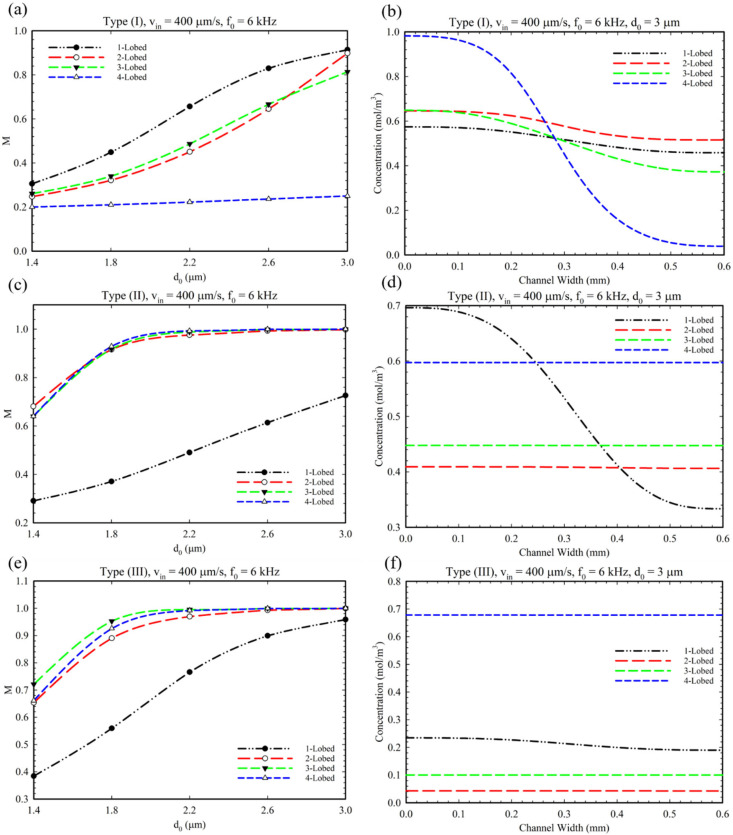
(**a**,**c**,**e**) The effect of the oscillation amplitude on the mixing for multi-lobed structures in Type I, II, and III configurations. (**b**,**d**,**f**) The related concentration profile at the microchannel outlet considering the effect of the change in the oscillation amplitude.

Figure 9 shows the effect of oscillation amplitude, $d_{0}$ (displacement of sharp-edge tip), on the mixing index for different conditions and configurations. This parameter represents the intensity of acoustic waves produced by piezoelectric actuators’ voltage. It is evident that by increasing the oscillation amplitude, the mixing index increased for all multi-lobed configurations. However, only for the 4-lobed structure of Type I configuration can we observe a slight increase in mixing index which can be explained because of the number of lobes and generating small vortices (Figure S3j). As explained in Figure 8e, we can observe a reduced concentration profile in Figure 9e for a similar reason. Additionally, we performed a comparative simulation study to clarify the effect of BAWs on the mixing performance of the Type III configuration by considering disabled and enabled acoustic modes (see Figures S5 and S6). As seen in these figures, the sole existence of obstacles does not have the ability to provide perfect mixing.

## 5. Chemical Reaction Analysis

**Figure 10:**
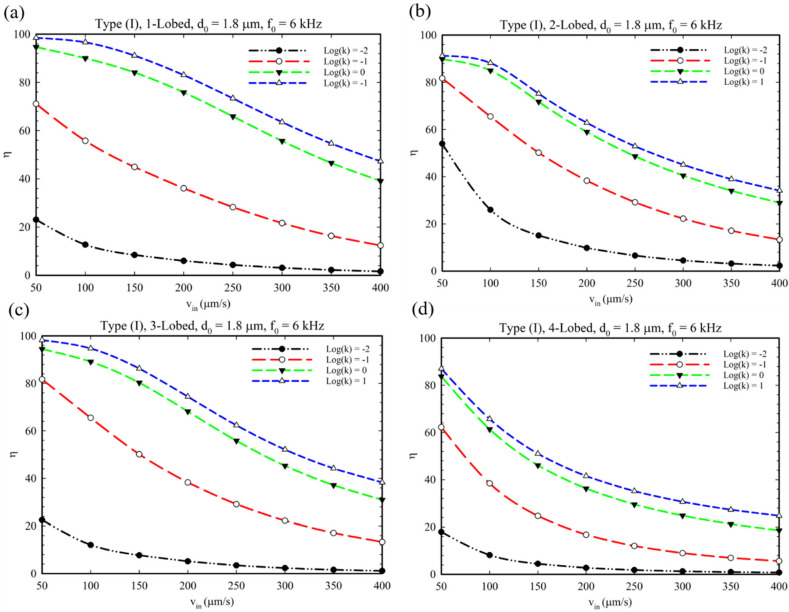
The reaction yield for chemical reaction at different fluid velocities for different multi-lobed structures and Type I configuration. (**a**–**d**) One to four-lobed structures.

**Figure 11:**
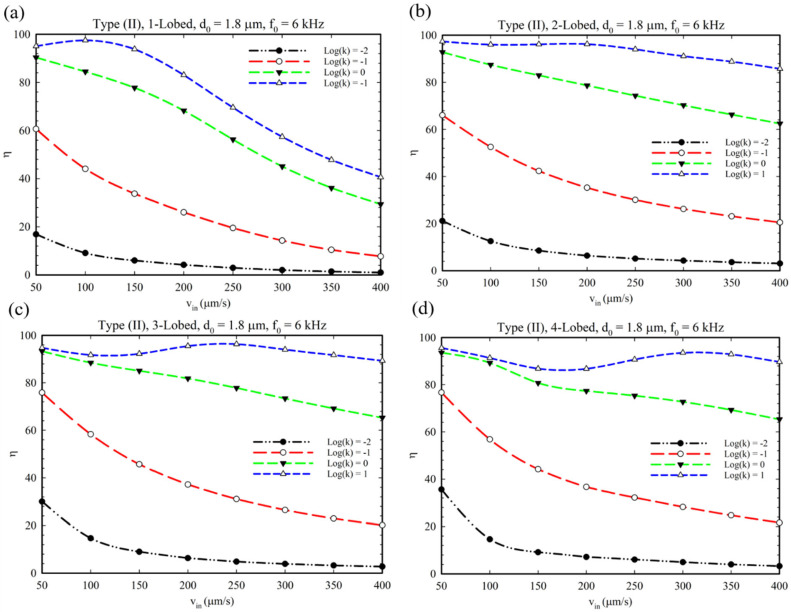
Production efficiency at different fluid velocities for type (II) microchannels with the geometries of (**a**) 1-lobed, (**b**) 2-lobed, (**c**) 3-lobed, and (**d**) 4-lobed structures.

**Figure 12:**
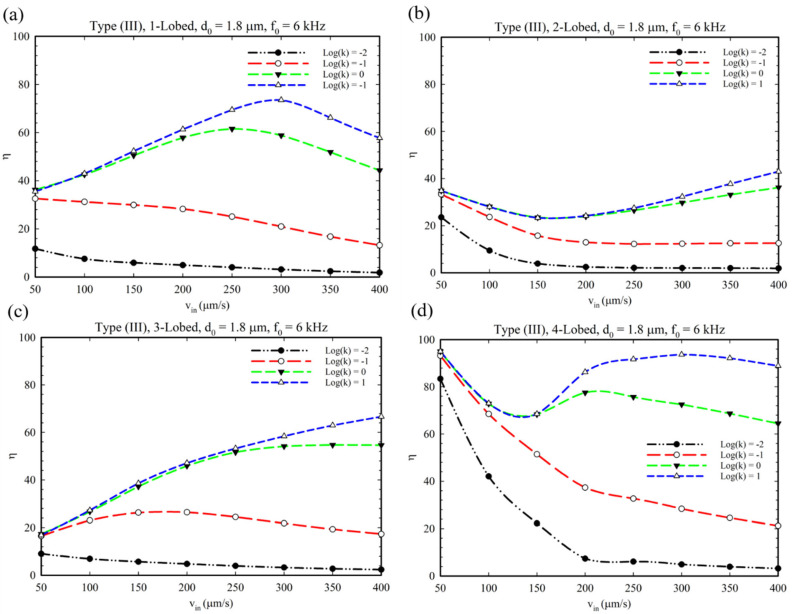
Production efficiency at different fluid velocities for type (III) microchannels with the geometries of (**a**) 1-lobed, (**b**) 2-lobed, (**c**) 3-lobed, and (**d**) 4-lobed structures.

In this section, we evaluate the performance of our acoustic micromixer for a chemical reaction. In this regard, an elementary, irreversible bimolecular reaction is considered (reaction Equation (5) in the Supplementary Materials) in which reactants (a and b) enter the reaction media separately via separate inlets. The effect of multi-lobed structures and different configurations are evaluated and shown in Figure 10, Figure 11 and Figure 12. The yield $\left(\eta_{i}\right)$ of the product substance in this reaction is calculated using Equation (2), which is defined as the ratio of the produced substance(s) to the initial value of reactants [38].

(2) $$\eta_{i}=\left(\frac{\int (C_{i}\left|\vec{v}\;\vec{.dA}\right|)}{\int (C_{0}\left|\vec{v}\;\vec{.dA}\right|)}\right)\times 100.$$

In this regard, Figure 10 demonstrates the micromixer’s behavior at different multi-lobed obstacles for Type I configuration. It is evident that by increasing the inlet velocity, the mixing index decreased, and the reaction yield decreased because the reactants did not have enough time to mix and perform the reaction. Additionally, the higher the reaction rate, the higher the expected reaction yield. Furthermore, as demonstrated in Figure 11, such behavior for reaction yield for the Type II configuration can be expected except for the reaction having k = 1. This observation proves that by increasing the reaction rate’s constant, this parameter can compensate for the fall in reaction yield due to the inlet velocity increase. This kind of (semi)linear trend due to alteration of the reaction yield in response to inlet velocity for multi-lobed obstacles is mainly caused by a proper mixing efficiency provided by this kind of acoustic sharp-edge configuration.

In Figure 12, the performance of the Type III configuration of the acoustic obstacle is demonstrated. As shown, similar to the Type II configuration, the 1, 2, and 3-lobed structures have semi-linear behavior due to the value of the reaction rate and the specific structure of oscillatory obstacle, which can resist the reducer factors (increasing in inlet velocity) (Figure 12a–c). Surprisingly, the yield of reaction revealed an ascending behavior for the higher value of the reaction rate constant (logk = 0, 1). This occurrence originates from two factors: (1) The structure of oscillatory obstacles, which produce vortices and a strong perturbation to establish an efficient mixing (See Figure S4). (2) The higher value of reaction rate constants provides a quick production of products. Considering these two synergic effects, the reaction yield experiences an ascending trend by increasing the inlet velocity. Adversely, for the 4-lobed structure (Figure 12d), a decreasing behavior in the graph can be recognized for lower values of the reaction’s rate. This issue may be attributed to the obstacle’s structure, which cannot provide proper mixing at higher values of inlet velocities because of producing small and weak vortices. However, for the higher values of the rate constant (=0, 1), it plays the dominant role, and for inlet velocities higher the 150 µm/s, the graph undergoes an ascending/descending behavior.

## 6. Conclusions

In summary, we analyzed the performance of an acoustically driven micromixer with leaf-shaped oscillatory obstacles as a nature-inspired scheme. In the first part, by considering multi-lobed structures and different placements for obstacles in the microchannel, the efficiency of these conditions was evaluated numerically. It was revealed that the mixing performance increased by increasing the number of lobes and decreasing their internal angle. Generally, we found that the sharper tips of lobes produce stronger vortices, making the mixing easier. Through our investigations, it was revealed that increasing the lobes inside the angle $\left(\alpha \right)$ from 15° to 45° for the Type I microchannel, a 16% decrease in mixing index was observed. Furthermore, in the Type II configuration, by increasing this parameter, a 28% increase in mixing performance was detected due to its particular design. Generally, higher values for pitch angle $\left(\beta \right)$ and lower values for inside angles $\left(\alpha \right)$ are preferred for multi-lobed obstacle structures. Furthermore, by increasing the length $\left(L_{E}\right)$ of lobes from 200 µm to 350 µm, 15% enhancement was recognized for the 1, 2, and 4-lobed structures of the Type I configuration. For the 4-lobed structure, no significant change was observed. Similarly, for the Type II configuration, increasing lobe length enhanced the mixing performance up to 99% percent, but for the longer than 300 µm, a decrease in concentration profile (≈6%) d. Additionally, it was found that increasing the inlet velocity from 50 up to 400 µm/s can reduce the mixing index to 60% for the Type I configuration (multi-lobes) and the Type II, 4-lobed system. Furthermore, it was proved that the acoustic waves’ frequency and amplitude are crucial to enhancing the mixing process, i.e., from 4 kHz to 10 kHz, we measured up to an 80% increase in mixing performance for all types of configurations of multi-lobed structures. Additionally, the displacement amplitude was identified as a considerable parameter and its change from 1.4 to 3 µm caused elevating of the MI from 0.25 to 0.9 for Type I 1, 2, and 3-lobed structures. In the second part, to analyze the chemical reaction in the microchannel, we found that two items of inlet velocity and reaction rate have prominent and competitive effects on the reaction yield. In these conditions, a decrease in the reaction yield by up to 50% was measured for Type I, multi-lobed structures for a 350 µm/s increase in inlet velocity. Additionally, the 3 and 4-lobed structure of the Type II configuration showed better performance, which was affected slightly by increasing the inlet velocity. It appeared that at higher values of inlet velocities (400 µm/s), the reactions with high rates (*Log*(*k*) = 1) could provide more appropriate production rates, i.e., the reaction yield experienced 40% enhancement for the 3-lobed Type III mode. Although acoustic micromixers have some limitations, such as requiring costly equipment and precise fabrication methods, they can perfectly mix substances in a small length of microchannels. Therefore, through these findings, we believe that such designing of acoustically driven micromixers can be exploited in various types of applications, from synthesizing controlled-shaped nanomaterials to diagnostics purposes at high efficiency and simplicity.

## Figures and Tables

**Table 1:** The boundary conditions used for simulation.

Type	Condition	Description
Fluid	$v_{0}$ = $v_{in}$	The velocity of fluid at inlets (zeroth order or background velocity)
	$v_{0}$ = 0	No-slip conditions on the channel’s walls
	$v_{1}$ = 0	First-order acoustic velocity (on the walls)
	$n.v_{1}$=$v_{bc}\left(y,z\right)e^{-i\omega t}$	On the oscillatory walls (Sharp-edges)
	$v_{2}$ = 0	On the walls
Thermal	$T_{\;}$ = $T_{0}$	On the walls
Concentration	$c_{\;}$ = 0	Concentration of solute at inlet 1
	c = 1 mol/m^3^	Concentration of solute at inlet 2
	cv = 0	No flux on the walls
	cv−D∇c=0	Outflow condition at outlet

**Table 3:** Optimized value for angles of α and θ.

θ	α	Structure of Lobes	Type
N/A	15	1-Lobed	Type (I)
80	15	2-Lobed	
120	15	3-Lobed	
120	35	4-Lobed	
N/A	45	1-Lobed	Type (II)
120	15	2-Lobed	
120	15	3-Lobed	
120	15	4-Lobed	

## Data Availability

The data presented in this study are available on request from the corresponding authors.

## Supplementary Materials

The following supporting information can be downloaded at: https://www.mdpi.com/article/10.3390/mi14040795/s1, Figure S1: Verification of our simulation; Figure S2: The graph for illustrating the mesh independent study; Figure S3: The illustration of generated vortices around oscillatory lobes for different structures and configurations; Figure S4: The distribution of reactants and products of chemical reaction through acoustic microchannel for different multi-lobed and configurations; Figure S5: The distribution of solute concentration; Figure S6: The concentration profile of solute along the microchannel width under disabled BAW conditions. References [27,35,40,41] are cited in the Supplementary Materials.
