# Supplementary material for: Investigating the Performance of the Multi-Lobed Leaf-Shaped Oscillatory Obstacles in Micromixers Using Bulk Acoustic Waves (BAW): Mixing and Chemical Reaction
Source: Micromachines (Basel). 2023 Mar 31;14(4):795. doi: 10.3390/mi14040795 (PMC10145993; doi:10.3390/mi14040795)
Supplement: Supplementary file 1 [file micromachines-14-00795-s001.zip › micromachines-2299377-supplementary.pdf]

Investigating the Performance of the Multi-Lobed Leaf-Shaped Oscillatory Obstacles in Micromixers Using Bulk Acoustic Waves (BAW): Mixing and Chemical Reaction

Vahid Kordzadeh-Kermani [1,†], Hossein Dartoomi [1,†], Mina Azizi [2], Seyed Nezameddin Ashrafizadeh [1,*] and Masoud Madadelahi [3,4,*]

1 Research Lab for Advanced Separation Processes, Department of Chemical Engineering, Iran University of Science and Technology, Tehran 16846-13114, Iran; v_kordzadeh@chemeng.iust.ac.ir (V.K.-K.)
2 Department of Electronics, South Tehran Branch Azad University, Tehran 1584715414, Iran
3 Department of Mechanical Engineering, Isfahan University of Technology, Isfahan 84156-83111, Iran
4 School of Engineering and Sciences, Tecnologico de Monterrey, Monterrey, NL 64849, Mexico
* Correspondence: ashrafi@iust.ac.ir (S.N.A.); masoud.m@tec.mx (M.M.)
† These authors contributed equally to this work.

Governing equations:

For the microfluidic geometries, which comprise the flow of fluids, the equations of motion for a laminar regime (continuity and momentum) are defined as equations (1) and (2):

$$\frac{\partial \rho}{\partial t} + \nabla.(\rho v) = 0 \tag{1}$$

$$\rho\frac{\partial v}{\partial t} + \rho(v.\nabla)v = -\nabla p + \mu\nabla^2 v + \left(\mu_b + \frac{1}{3}\mu\right)\nabla(\nabla.v) \tag{2}$$

where the parameter ρ is density, v is velocity, p is the pressure of the fluid, μ is the dynamic viscosity, and μ_b is the bulk viscosity of the fluid [1]. Additionally, the pressure in the fluid can be defined as a function of sound speed in liquid (equation 3) in which c_0^2 is the speed of sound in the liquid [2].

$$p = c_0^2\rho \tag{3}$$

Since it occurs in the mass transfer system, the equation for representing mass transport can be written as equation (4), where c_i is the concentration of species i, J_i represents molecular mass flux, and R_i is the expression for reaction rate.

$$\frac{\partial c_i}{\partial t} + \nabla.J_i + v.\nabla c_i = R_i \tag{4}$$

For the chemical reaction, we considered a bimolecular irreversible elementary chemical reaction with a variable reaction rate constant as shown in equations (5) and (6).

$$a + b \xrightarrow{k} c \tag{5}$$

$$R_i = kC^aC^b \tag{6}$$

By considering appropriate boundary conditions and combining the above-mentioned equations, the movement of fluid in the microfluidic device can be solved numerically.

(a)

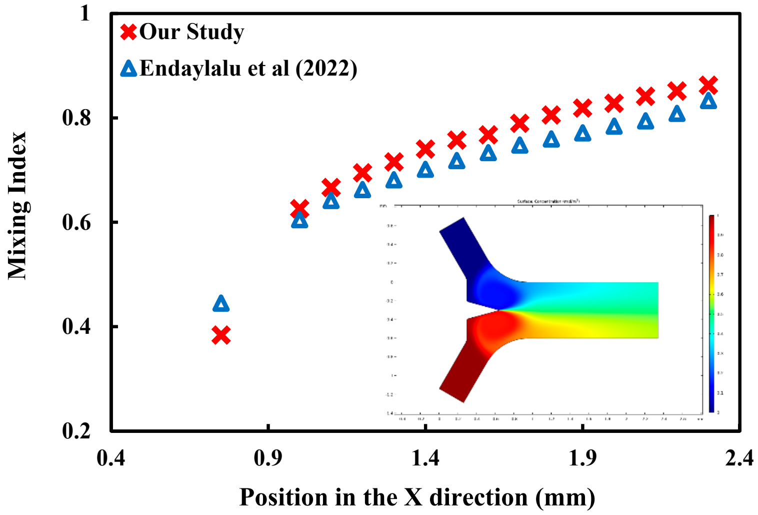

(b)

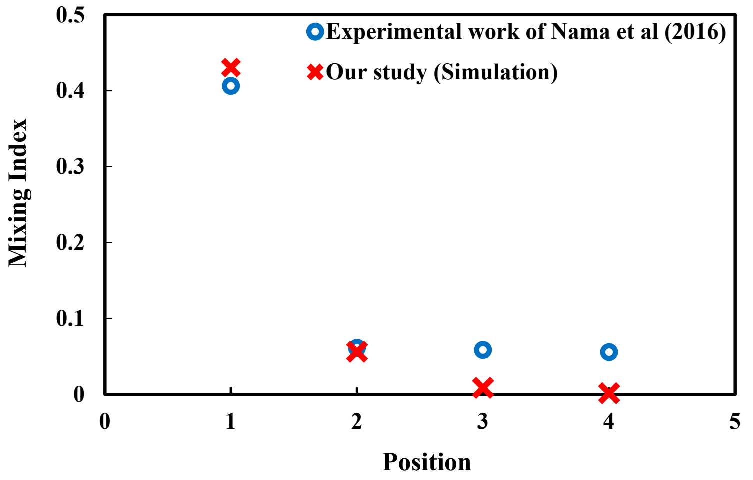

Figure S1. (a) Verification of our simulation vs the numerical work of Endaylalu and Tien (2022) [3] mean error ≈ 5% (inlet velocity = 55.6 µm/s) (MI = 1 represents perfect mixing). (b) The validation of our simulation results with the experimental works of Nama et. al at different length positions of microchannel (Q_{in} = 2 μl/min), position 1: y = 1100 μm, position 2: y = 1700 μm, position 3: y = 2300 μm, and position 4: y = 2900 μm (MI = 0 represents perfect mixing and MI = 0.5 represents unmixed fluids) [4].

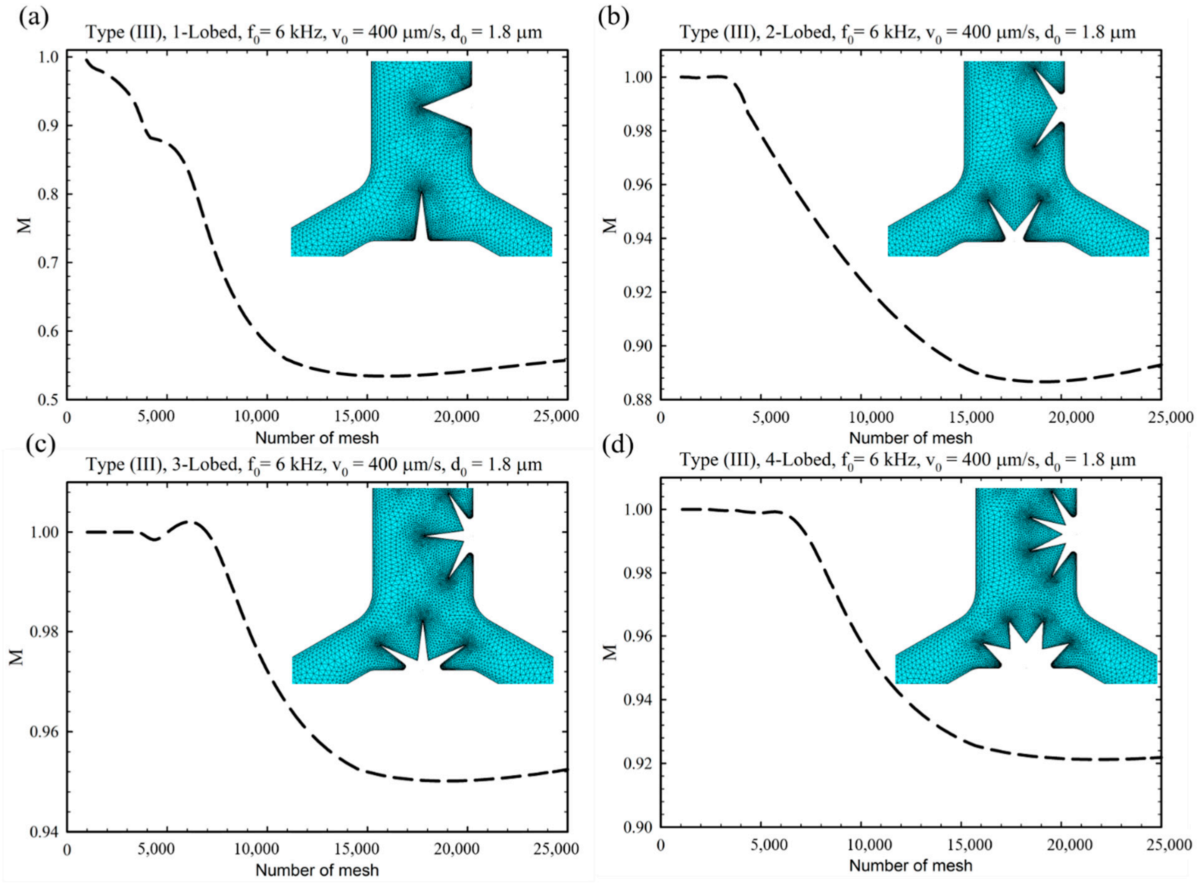

Figure S2. The graph for illustrating the mesh independent study. (a) 1-lobed, (b) 2-lobed, (c) 3-lobed, and (d) 4-lobed structures.

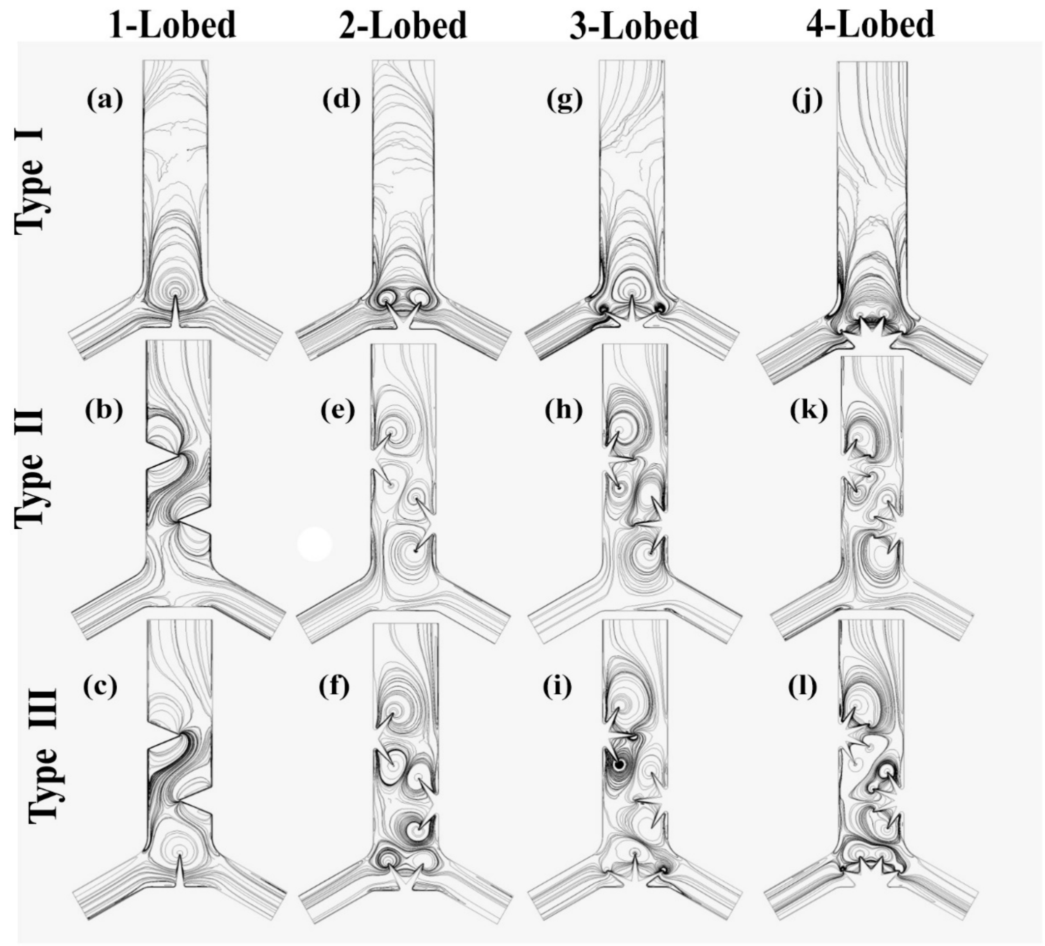

Figure S3. The illustration of generated vortices around oscillatory lobes for different structures and configurations. (**a,b,c**) 1-lobed structure in Type (I, II, and III) configurations, (**d,e,f**) 2-lobed structure in Type (I, II, and III) configurations, (**g,h,i**) 3-lobed structure in Type (I, II, and III) configurations, and (**j,k,l**) 4-lobed structure in Type (I, II, and III) configurations.

Type III

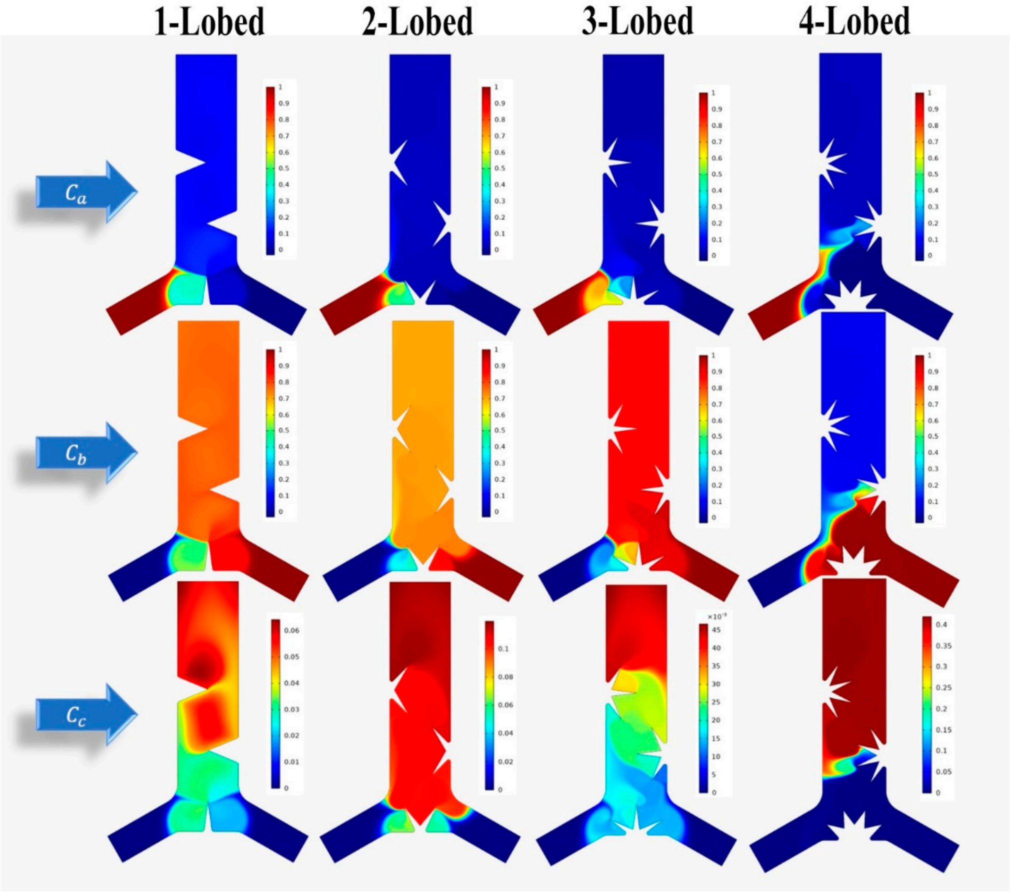

Figure S4. The concentration distribution of reactants (C_a and C_b) and products (C_c) of chemical reaction through acoustic microchannel for different multi-lobed and configurations.

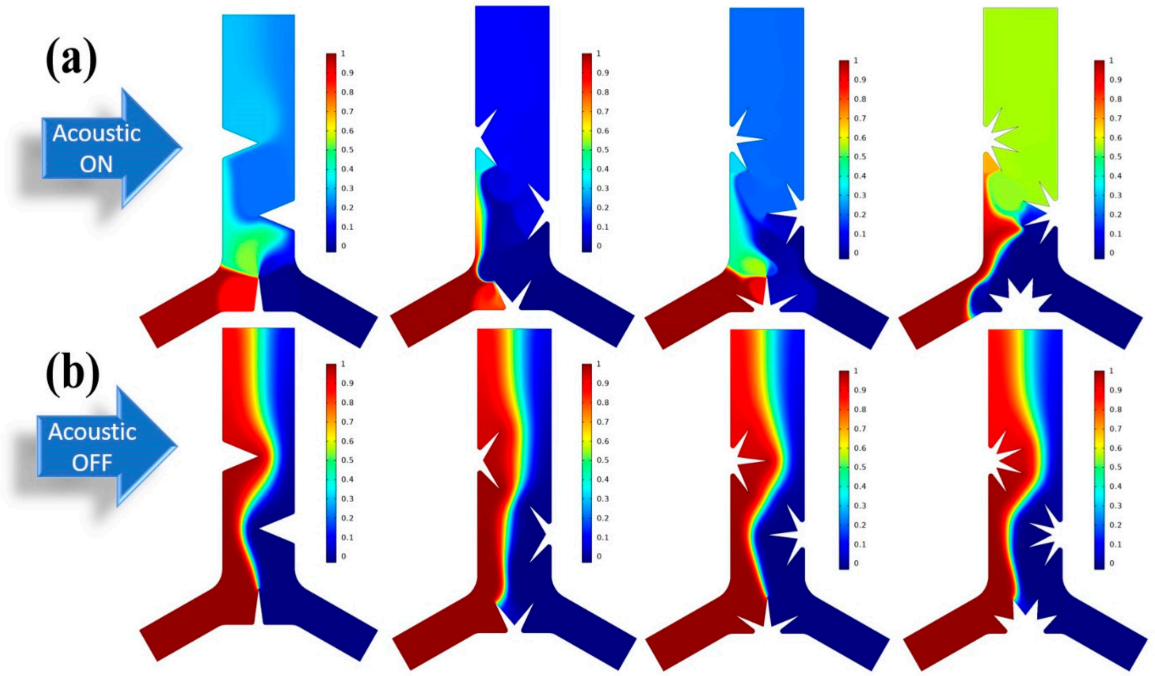

Figure S5. The distribution of solute concentration by considering (a) enabled and (b) disabled acoustic waves.

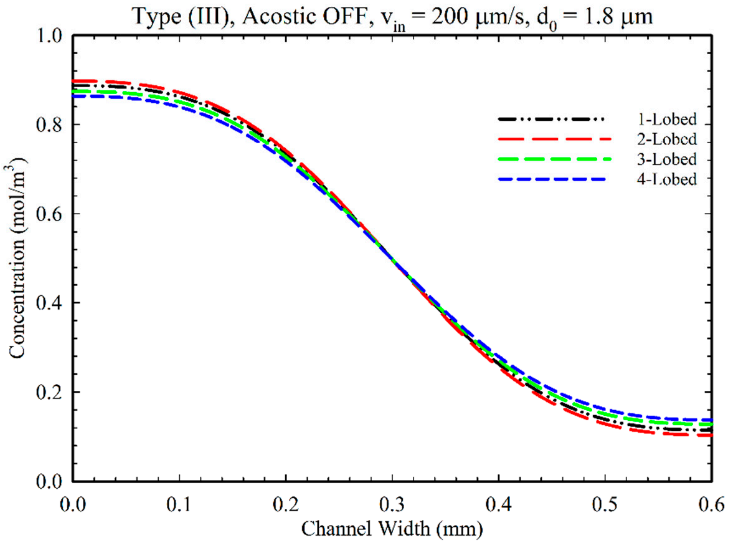

Figure S6. The concentration profile of solute along the microchannel width under disabled BAW conditions (Type III configuration, 1, 2, 3, 4-lobed structures, inlet velocity = 200 μm/s)

Reference

1. Roberson, J.A.; Elger, D.F.; Crowe, C.T. Engineering fluid mechanics. **2019**.
2. Nama, N.; Barnkob, R.; Mao, Z.; Kähler, C.J.; Costanzo, F.; Huang, T.J. Numerical study of acoustophoretic motion of particles in a PDMS microchannel driven by surface acoustic waves. *Lab Chip* 2015, *15*, 2700–2709, doi:10.1039/c5lc00231a.
3. Endaylalu, S.A.; Tien, W.H. A Numerical Investigation of the Mixing Performance in a Y-Junction Microchannel Induced by Acoustic Streaming. *Micromachines* **2022**, *13*, doi:10.3390/mi13020338.
4. Nama, N.; Huang, P.H.; Huang, T.J.; Costanzo, F. Investigation of micromixing by acoustically oscillated sharp-edges. *Biomicrofluidics* 2016, *10*, doi:10.1063/1.4946875.
